# Assessing the contribution of genetic nurture to refractive error

**DOI:** 10.1038/s41431-022-01126-6

**Published:** 2022-05-27

**Authors:** Jeremy A. Guggenheim, Rosie Clark, Tetyana Zayats, Cathy Williams, the UK Biobank Eye and Vision Consortium, Jeremy A. Guggenheim, Cathy Williams

**Affiliations:** 1 School of Optometry & Vision Sciences, Cardiff University, Cardiff, CF24 4HQ UK; 2 Analytic and Translational Genetics Unit, Department of Medicine, Massachusetts General Hospital and Harvard Medical School, Boston, MA 02114 USA; 3 Stanley Center for Psychiatric Research, Broad Institute of MIT and Harvard, Cambridge, MA 02142 USA; 4 PROMENTA, Department of Psychology, University of Oslo, Oslo, Norway; 5 Centre for Academic Child Health, Population Health Sciences, Bristol Medical School, University of Bristol, Bristol, BS8 2BN UK; 6 School of Optometry & Vision Sciences, Cardiff University, Cardiff, CF24 4HQ UK; 7 Centre for Academic Child Health, Population Health Sciences, Bristol Medical School, University of Bristol, Bristol, BS8 2BN UK

**Keywords:** Risk factors, Diseases

## Abstract

Parents pass on both their genes and environment to offspring, prompting debate about the relative importance of nature versus nurture in the inheritance of complex traits. Advances in molecular genetics now make it possible to quantify an individual’s genetic predisposition to a trait via his or her ‘polygenic score’. However, part of the risk captured by an individual’s polygenic score may actually be attributed to the genotype of their parents. In the most well-studied example of this indirect ‘genetic nurture’ effect, about half the genetic contribution to educational attainment was found to be attributed to parental alleles, even if those alleles were not inherited by the child. Refractive errors, such as myopia, are a common cause of visual impairment and pose high economic and quality-of-life costs. Despite strong evidence that refractive errors are highly heritable, the extent to which genetic risk is conferred directly via transmitted risk alleles or indirectly via the environment that parents create for their children is entirely unknown. Here, an instrumental variable analysis in 1944 pairs of adult siblings from the United Kingdom was used to quantify the proportion of the genetic risk (‘single nucleotide polymorphism (SNP) heritability’) of refractive error contributed by genetic nurture. We found no evidence of a contribution from genetic nurture: non-within-family SNP-heritability estimate = 0.213 (95% confidence interval 0.134–0.310) and within-family SNP-heritability estimate = 0.250 (0.152–0.372). Our findings imply the genetic contribution to refractive error is principally an intrinsic effect from alleles transmitted from parents to offspring.

## Introduction

**Fig. 1:**
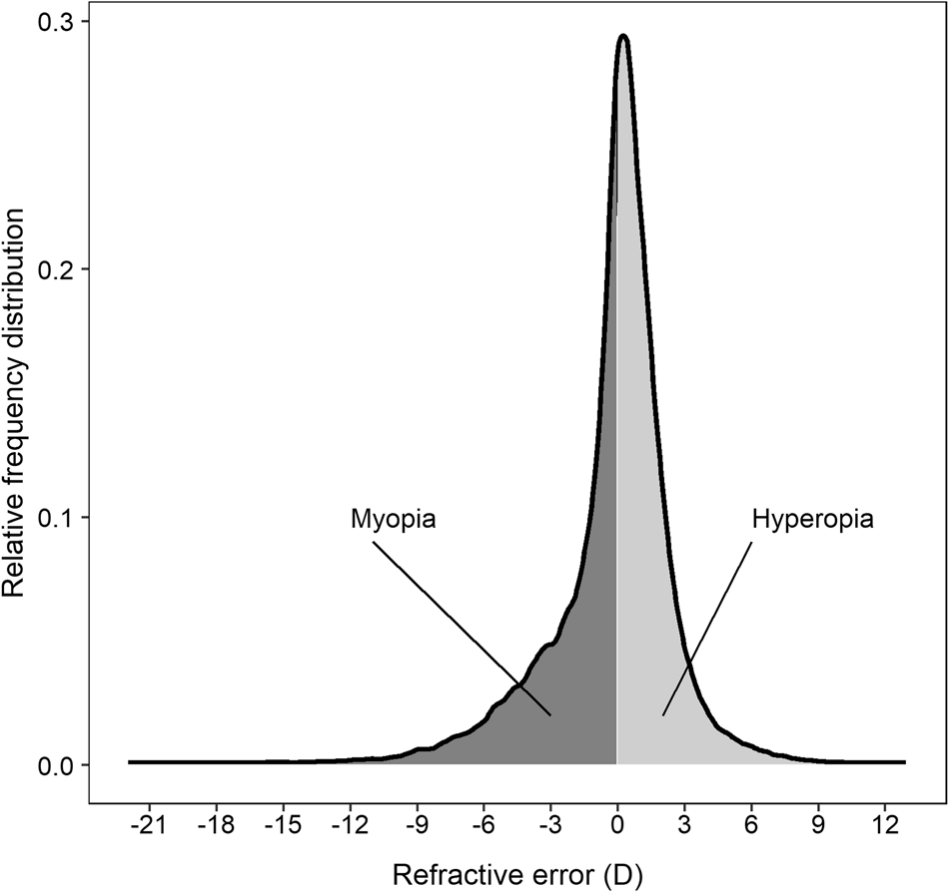
Characteristics of the refractive error distribution and its relationship to myopia and hyperopia. Myopia represents the negative arm and hyperopia the positive arm of the refractive error distribution. Data are from adult UK Biobank participants of European ancestry.

A mismatch between the focal and axial lengths of the eye causes blurry vision, known as myopia or hyperopia (Fig. 1). Approximately 30–50% of children in the USA and Europe and 50–90% of children in East and South-East Asia develop myopia by early adulthood [1, 2]. Refractive errors impose a heavy economic burden on society in terms of the need for sight tests and visual correction (glasses, contact lenses or refractive surgery). Myopia also imposes a healthcare and societal burden via an increased risk of visual impairment and blindness, through myopic macular degeneration, retinal detachment and a heightened risk of glaucoma and cataract [3].

Refractive error is a highly heritable trait [4–7], with most cases of myopia developing when genetically susceptible individuals are exposed to environmental risk factors [8]. To date, insufficient time outdoors and intensive education are regarded as major risk factors for myopia [1, 9–12]. The interplay between genetic and lifestyle risk factors is a major focus of current research efforts in refractive error [13–15].

### Genetic nurture

**Fig. 2:**
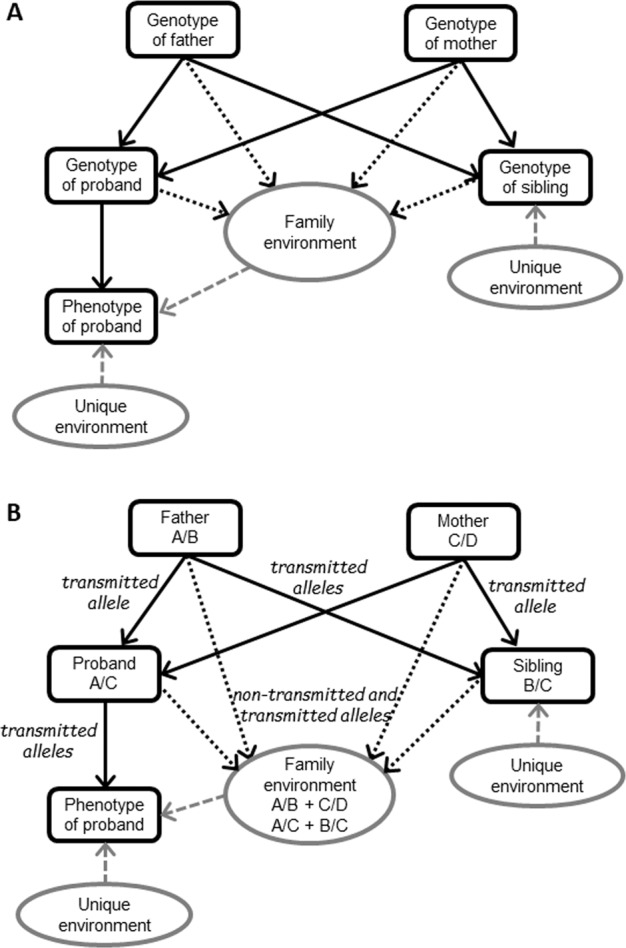
Path diagram illustrating how transmitted and non-transmitted alleles may contribute to a child’s phenotype. Solid black arrows indicate causal pathways acting via direct transmission of risk alleles (direct genetic effects). Dotted black arrows indicate causal pathways of genotypes acting on the environment (indirect genetic effects, genetic nurture). Grey dashed arrows indicate causal pathways acting via the environment. For simplicity, some potential causal pathways are omitted. Panel **A** illustrates the key principles. Panel **B** illustrates an example for a single hypothetical multiallelic genetic marker, such as a microsatellite, with 4 alleles. The proband has inherited alleles A and C from the father and mother, respectively. However, as well as these transmitted alleles (with both direct and indirect genetic effects), the non-transmitted parental alleles B and D may also contribute to the nurturing behaviour of the parents (indirect genetic effects). Parental alleles B and C have been inherited by the proband’s sibling. These alleles–one of which was not transmitted to the proband–may nevertheless influence the phenotype of the proband, e.g. by influencing the environment of the sibling (indirect genetic effect).

The genetic risk transmitted from parents to offspring generally occurs via the transmission of parental alleles to children. However, alleles present in the parents can potentially influence the phenotype of a child through the child’s environment, independently of their transmission to the child (‘genetic nurture’) [16]. Accordingly, children may ‘inherit both phenotype-associated SNPs and phenotype-associated environments from parents’ [17]. Figure 2 illustrates how the family environment of a child (the proband) can be influenced by the proband’s own genotype and the genotypes of the two parents and sibling(s).

A genetic variant displaying a significant genotype-phenotype association provides evidence of a causal path affecting the phenotype [18]. However, in the context of genetic nurture, it is apparent that this causal path could act partly or fully via the environment of a proband (which is affected by the genetics of his/her relatives). The presence of genetic nurture may have profound implications. For instance, when aiming to translate discoveries from genome-wide association studies (GWASs) into treatments, it is critical to determine whether disease-associated SNPs act within a proband or through his/her environment. If the former is true, then an intervention (such as a drug) would need to be targeted at the proband, while if the latter is true, an intervention may need to be targeted at the proband’s environment.

To date, the role of genetic nurture has only been examined for a few traits. Most studies have focused on either educational attainment or birth weight [16, 19–28]. These studies have demonstrated that as much as half of the genetic contribution to educational attainment may arise via genetic nurture rather than via transmitted alleles [16, 20, 22–26]. This is suggestive, for example, that alleles carried by the parents and influencing their own educational attainment [29] may lead them to influence the development of educational attainment in children by placing more or less than average emphasis on their child’s academic environment, e.g. via encouragement to study or provision of resources, such as books and private tuition. In the case of birth weight, several genome-wide significantly associated variants have been shown to act via the foetal environment [30].

### Polygenic scores and SNP-heritability

A polygenic score (PGS) quantifies a person’s genetic predisposition for a specific trait [31] and can be harnessed to provide an unbiased estimate of the SNP-heritability by implementing a ‘split-sample’ GWAS approach [32, 33]. This involves dividing the available GWAS sample at random into two equally sized groups of participants and running a GWAS in each subsample, thereby yielding two independent estimates of the SNP effect sizes (beta coefficients). An unbiased estimate of the SNP-heritability can then be obtained by using one of the resultant polygenic scores as an instrumental variable for the other [32, 33].

When a GWAS is carried out in a sample of unrelated individuals, the SNP beta coefficients will not only capture effects of transmitted alleles, but also any effects due to genetic nurture, population stratification and assortative mating. By contrast, an appropriately designed *family-based* GWAS will only capture the genetic effects of transmitted alleles. Here, we carried out analyses to assess the evidence for genetic nurture by comparing SNP-heritability estimates obtained in within-family vs. non-within-family analyses [21]. Given the evidence for a causal role of education in predisposing children to myopia [11, 12, 34], we hypothesized that genetic nurture would make a major contribution to the SNP-heritability of refractive error. We also performed the same analysis for a trait known to show genetic nurture, educational attainment.

## Methods

### Participants and phenotypes

UK Biobank is a prospective study of health and well-being of adults living in the United Kingdom. Ethical approval for the study was obtained from the NHS Research Ethics Committee (Reference: 11/NW/0382) and all participants provided signed, informed consent. Recruitment occurred during 2006–2010, when approximately 500,000 individuals aged 37–73 years attended the baseline assessment visit [35]. At this visit, participants completed a structured interview that included the question, “Which of the following qualifications do you have? (You can select more than one)”. A follow-up question was asked of those who did not report having a university or college degree, “At what age did you complete your continuous full-time education?”. Educational attainment (*EduYears)* was defined as age participants reported completing their full-time education (minus 5 years, which is the age the participants started formal schooling), except that those who reported leaving school before age 15 or after age 21 years-old were assigned a school leaving age of 15 or 21 (thus, an *EduYears* of 10 or 16 years), respectively [12]. Participants with a university or college degree were assigned a school leaving age of 21 (an *EduYears* of 16 years). An ophthalmic assessment was introduced late in the UK Biobank recruitment period, with only about a quarter of participants undergoing this assessment. The mean spherical equivalent (sphere plus 0.5 × cylinder) for each eye was calculated from the autorefraction readings. The average mean spherical equivalent (*avMSE*) of the two eyes was taken as the participant’s refractive error phenotype value [12].

### Selection of siblings for genetic nurture analysis and participants for split-sample genome-wide association studies

**Fig. 3:**
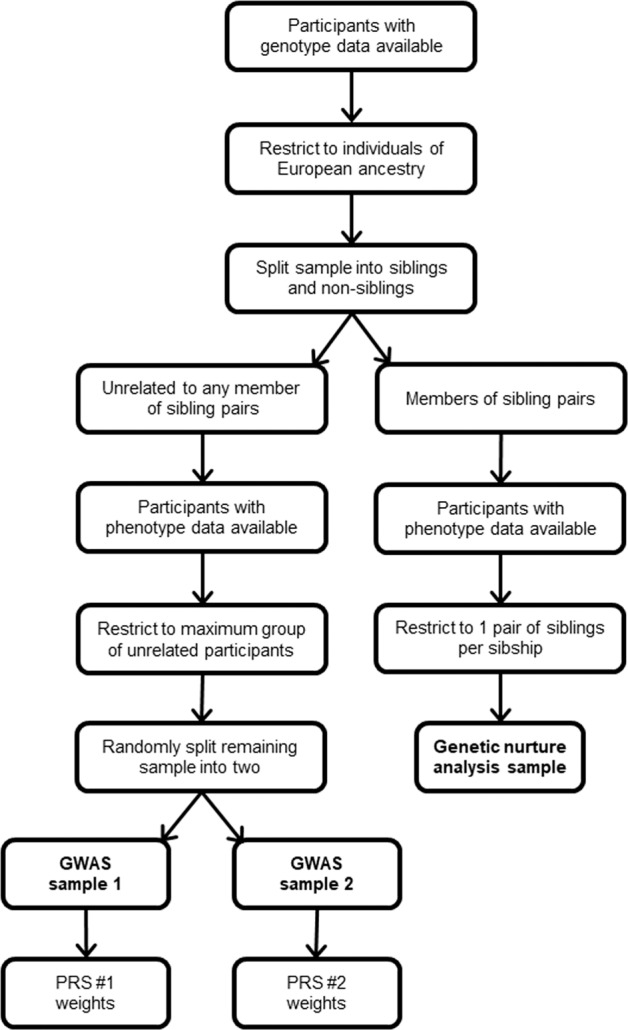
Selection scheme for genetic nurture analysis and split-sample genome-wide association studies.

An overview of the selection scheme is shown in Fig. 3. All analyses were restricted to participants of European ancestry. Siblings were identified as described by Bycroft et al. [36]. Two outlier pairs whose kinship vs. identity-by-state allele sharing pattern did not cluster with the other sibling pairs were excluded (Supplementary Fig. S1). For sibships with more than 2 individuals with information on the traits of interest, we randomly selected one pair. We refer to this sample of siblings as the ‘genetic nurture analysis sample’ (Fig. 3). For the split-sample GWASs for each trait-of-interest, we selected participants who were unrelated to any individual in the genetic nurture analysis sample and who were also unrelated to one another (Fig. 3), using the R package *igraph* (Bycroft et al. [36]). The pool of available participants for the split-sample GWAS analyses was divided at random into two equally sized groups. The sample size for the split-sample GWAS for *avMSE* was 2 × *n* = 43,168 and for *EduYears* was 2 × *n* = 147,813.

### Genome-wide association (GWA) analyses and polygenic scores

A list of autosomal genetic variants in the HapMap3 reference panel was downloaded from the LDSC website [37] (https://alkesgroup.broadinstitute.org/LDSCORE/w_hm3.snplist.bz2). Using PLINK v2.00a2LM [38], imputed genotype data from UK Biobank release 3 were filtered to retain only variants present on the HapMap3 list that had a missing genotype rate <0.02, restricted to participants of European ancestry with a missing genotyping rate <0.05. The variants were sorted in descending order of minor allele frequency (MAF) and the top 999,998 variants were selected for inclusion in the genome-wide association studies (these retained variants all had MAF > 0.02). GWA analyses were performed separately for split sample 1 and split sample 2 for each trait of interest (*n* = 43,168 for *avMSE* and *n* = 147,813 for *EduYears*) using the --predBetasFile option in BOLT v2.3.5 [39]. Age, age-squared, sex, genotyping array and the first 10 genetic principal components (PCs) were included as covariates. The above GWA analyses were performed for an infinitesimal model. Polygenic scores for the genetic nurture samples were calculated using the PLINK v1.9 --score function, with the weights obtained from BOLT. In total, four polygenic scores were calculated: two polygenic scores for *avMSE* and two polygenic scores for *EduYears*. The incremental R^2^ was defined as the variance in the trait-of-interest explained by the polygenic score in a linear regression model, over and above the variance explained by a baseline linear regression model that included the predictors age, age-squared, sex, genotyping array and the first 10 genetic PCs.

### Assessment of genetic nurture

Each participant’s genetic propensity for the trait of interest was quantified using a polygenic score (PGS), using Eq. (1).

1 $${{{{{\rm{PGS}}}}}} = {\sum} {\beta _{jk}}$$

where, *β*_*j*_ is a vector of weights for a set of *J* genetic variants indexed by *j* = 1, 2, … *J* and *k* = 0, 1 or 2 is the number of effect alleles for variant *j* carried by the participant. The weights *β*_*j*_ are obtained in a GWAS for the trait of interest, after accounting for linkage disequilibrium (LD). The aim of the current study was to examine the extent to which the genetic risk captured by a polygenic score for refractive error is mediated by genetic nurture effects. In this context, use of an imprecise polygenic score will tend to underestimate the contribution of the mediating pathway [31]. One method to avoid this source of bias is to estimate $\widehat {\beta _j}$ using a split-sample GWAS [32, 33]. This yields two independent estimates of the genetic variant weights $\widehat {\beta _j}$ and an unbiased estimate of PGS can be obtained by using one of the $\widehat {\beta _j}$ estimates as an instrumental variable for the other [32, 33].

We fitted Eq. (2), following Brumpton et al. [40].

2 $$y_{fi} = \alpha _0 + \alpha _1\widehat {{{{{\rm{PGS}}}}}}_{(m = 2)fi} + W_f + u_{fi}$$

$${{{{{\rm{PGS}}}}}}_{\left( {m = 2} \right)fi} = {{{{{\rm{PGS}}}}}}_{\left( {m = 1} \right)fi} + v_{fi}$$

where, *α*_0_ is the mean phenotype level in the sample, *y*_*fi*_ is the phenotype of sibling *i* = A or B from family *f* who has an instrumented polygenic score of $\widehat {{{{{\rm{PGS}}}}}}_{(m = 2)fi}$, and *W*_*f*_ is the mean level of the phenotype in family *f*. Terms ${{{{{\rm{PGS}}}}}}_{\left( {m = 1} \right)fi}$and PGS_(*m*=2)*fi*_ are the polygenic scores derived from the first and second tranches of a split-sample GWA, in which the first polygenic score is used as an instrumental variable for the second. Age, age-squared, sex, genotyping array and the first 10 principal components (PCs) derived by Bycroft et al. [36] were included in Eq. (2). The parameter *α*_1_ in Eq. (2) is a within-family measure of the genetic contribution to the trait of interest, which we refer to as: $\alpha _1^{wf}$. An equivalent model can also be fitted for a sample of unrelated individuals comprising of one sibling selected at random from each family (without including terms for family effects). This estimates the total contribution of the polygenic score to the phenotype ($\alpha _1^{nwf}$), by capturing both within-family and between-family effects. A difference in the magnitude of $\alpha _1^{wf}$ vs. $\alpha _1^{nwf}$ provides evidence for genetic nurture (and/or assortative mating and population stratification). Models were fitted in R v3.6.3, using the packages *plm* and *ivmodel* (code in Supplementary Methods).

## Results

Table 1 presents the demographic characteristics of the two samples of siblings used to assess the role of genetic nurture in educational attainment and refractive error. Table 1 also presents the demographic characteristics of the split-sample GWAS samples used to derive polygenic scores for each trait. A much larger sample size was available for the educational attainment analyses compared to the refractive error analyses, as autorefraction was performed only at the latter stages of UK Biobank recruitment.

**Table 1:** Demographic characteristics of participants. Values are presented as mean (95% confidence interval).

	Trait of interest: educational attainment (*EduYears*)			Trait of interest: refractive error (*avMSE*)		
Trait	Siblings sample *n* = 36,292	GWAS split sample 1 *n* = 147,813	GWAS split sample 2 *n* = 147,814	Siblings sample *n* = 3888	GWAS split sample 1 *n* = 43,169	GWAS split sample 2 *n* = 43,170
Female (proportion)	0.58 (0.57–0.58)	0.54 (0.53–0.54)	0.53 (0.53–0.54)	0.58 (0.56–0.59)	0.53 (0.53–0.53)	0.53 (0.52–0.53)
Age (years)	57.55 (57.48–57.63)	57.28 (57.24–57.32)	57.28 (57.24–57.33)	57.80 (57.57–58.04)	57.69 (57.62 to 57.76)	57.74 (57.67–57.82)
Height (cm)	168.07 (167.98 to 168.17)	168.99 (168.94 to 169.03)	168.98 (168.93–169.03)	168.25 (167.97–168.53)	169.21 (169.13–169.3)	169.26 (169.17–169.35)
TDI	−1.50 (−1.53 to −1.47)	−1.53 (−1.54 to −1.51)	−1.52 (−1.54 to −1.51)	−1.38 (−1.47 to −1.29)	−1.35 (−1.37 to −1.32)	−1.34 (−1.36 to −1.31)
University/College degree (proportion)	0.30 (0.29–0.30)	0.34 (0.34–0.35)	0.34 (0.34–0.35)	0.28 (0.26–0.29)	0.37 (0.37–0.38)	0.37 (0.36–0.37)
*EduYears* (years)	12.84 (12.81–12.86)	13.14 (13.12–13.15)	13.12 (13.1–13.13)	12.77 (12.7–12.85)	13.30 (13.28–13.33)	13.27 (13.24–13.29)
*avMSE* (D)^a^	–	–	–	−0.15 (−0.24 to −0.07)	−0.26 (−0.28 to −0.23)	−0.25 (−0.27 to −0.22)

*TDI* Townsend deprivation index (Z-score; higher positive values indicate greater social material deprivation).

^a^Refractive error measures were not available for the majority of the Educational Attainment samples.

### Contribution of genetic nurture to educational attainment

Split-sample GWAS analyses for *EduYears* were carried out in two non-overlapping samples of UK Biobank participants (Fig. 3). From the two sets of GWAS summary statistics, two independent polygenic scores for *EduYears* were derived. The variance in *EduYears* explained by each of these polygenic scores was assessed in a sample comprising of one randomly chosen sibling (sibling ‘A’) from the genetic nurture analysis sample for *EduYears*, which comprised of 18,146 sibling pairs who were unrelated to the participants in the GWAS samples. Each polygenic score explained approximately 8% of the variance in *EduYears*. Specifically, the incremental *R*^2^ for the first polygenic score PGS_(*m*=1)_ was *R*^2^ = 0.077 and for the second polygenic score PGS_(*m*=2)_ it was *R*^2^ = 0.080. The correlation of the two polygenic scores (PGS_(*m*=1)_ vs. PGS_(*m*=2)_ in sibling A) in this sample was 0.509 (95% CI 0.498–0.519).

For the first polygenic score PGS_(*m*=1)_ the correlation of the polygenic score for sibling A vs. sibling B in each sibship, i.e. the within-sibling-pair correlation, was 0.556 (95% CI 0.546–0.566). For PGS_(*m*=2)_ the corresponding within-sibling-pair correlation was 0.553 (95% CI 0.542–0.563). These within-sibling-pair correlations were above the level of 0.5 expected under random mating, suggesting the presence of assortative mating, as reported previously for the trait *EduYears* [41].

**Fig. 4:**
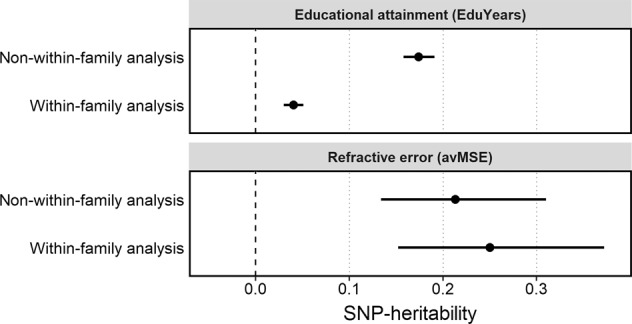
SNP-heritability of educational attainment (*EduYears*) and refractive error (*avMSE*) assessed using a within-family and non-within-family analysis. Error bars represent 95% confidence interval (these confidence intervals are asymmetric due to conversion from standardized regression coefficient scale to heritability scale).

The results of the genetic nurture analysis for *EduYears* are shown in Table 2, Fig. 4 and Supplementary Table S1. For probands in a sample composed of one randomly selected sibling from each pair, the instrumented polygenic score yielded an estimate of the SNP-heritability of $\alpha _1^{nwf}$= 0.174 (95% CI 0.158–0.191). By contrast, a within-family analysis provided a lower estimate, $\alpha _1^{wf}$ = 0.040 (95% CI 0.030–0.051). The relatively lower magnitude of $\alpha _1^{wf}$ compared to $\alpha _1^{nwf}$ (0.040 vs. 0.174) indicated that the contribution to the SNP-heritability of *EduYears* from genetic nurture and assortative mating combined was larger than the contribution from transmitted alleles. This is consistent with previous work, which has suggested that as much as half of the variance in educational attainment captured by a polygenic score acts via alleles present in the parents irrespective of transmission to the proband or reflects assortative mating.

**Table 2:** SNP-heritability ($h_{{{{{\rm{SNP}}}}}}^2$) of educational attainment and refractive error calculated in within-family and non-within-family analyses.

	Trait of interest: educational attainment (*EduYears*)			Trait of interest: refractive error (*avMSE*)		
*Analysis method*	$h_{{{{{\rm{SNP}}}}}}^2$	95% CI	*P* value	$h_{SNP}^2$	95% CI	*P* value
Non-within-family $(\alpha _1^{nwf})$	0.174	(0.158–0.191)	<1.00E−100	0.213	(0.134–0.310)	6.36E−21
Within-family $(\alpha _1^{wf})$	0.040	(0.030–0.051)	1.15E−47	0.250	(0.152–0.372)	1.32E−18

*95% CI* 95% confidence interval.

### Contribution of genetic nurture to refractive error

Following the same methodology described above for studying the contribution of genetic nurture to *EduYears*, split-sample GWAS analyses for *avMSE* were carried out in two further non-overlapping samples of UK Biobank participants (Fig. 3). From the two sets of GWAS summary statistics, two independent polygenic scores for *avMSE* were derived. The variance in *avMSE* explained by each of the polygenic scores was assessed in a sample comprising of one randomly chosen sibling from the genetic nurture analysis sample for *avMSE*, which comprised of 1944 sibling pairs. Each polygenic score for *avMSE* explained ~6% of the variance in *avMSE* in this independent sample. Specifically, the incremental R^2^ = 0.064 for PGS_(*m*=1)_ and R^2^ = 0.059 for *PGS*_(*m*=2)_ (*n* = 1944). The correlation of the two polygenic scores for *avMSE* (PGS_(*m*=1)_ vs. PGS_(*m*=2)_ in sibling A) in this sample of unrelated individuals was 0.316 (95% CI 0.275–0.355).

In the sample of *n* = 1944 sibling pairs with information on *avMSE*, the within-sibling-pair correlation of *PGS*_(*m*=1)_ was 0.477 (95% CI 0.442–0.511) and the within-sibling-pair correlation for *PGS*_(*m*=2)_ was 0.499 (95% CI 0.465–0.532). These correlations were close to the value of 0.5 expected under random mating, although the confidence intervals were wide. To provide more precise estimates, the within-sibling-pair analyses were repeated in the larger sample of siblings (*n* = 18,146 pairs) used for the genetic nurture analysis of EduYears. (Only genetic data were required to calculate the within-sibling correlation in polygenic scores for *avMSE*. Therefore, this calculation was possible even though information for the *avMSE* phenotype was mostly not available in this sample of 18,146 sibling pairs). Calculated for this sample of 18,146 sibling pairs, the within-sibling-pair correlation in polygenic scores for *avMSE* was 0.526 (95% CI 0.516–0.537) for PGS_(*m*=1)_ and 0.525 (95% CI 0.514 –0.535) for PGS_(*m*=2)_. These more precise estimates were indicative of assortative mating, although to a lesser degree than for *EduYears*.

An instrumented polygenic score for *avMSE* yielded a non-within-family SNP-heritability estimate of $\alpha _1^{nwf}$= 0.213 (95% CI 0.134–0.310) and a within-family estimate of $\alpha _1^{wf}$= 0.250 (95% CI 0.152–0.372). Notably, the within-family SNP-heritability estimate for *avMSE* was not lower than the non-within-family estimate (0.213 vs. 2.50; Table 2, Fig. 4 and Supplementary Table S2). This lack of a reduction in the within-family estimate suggested that genetic nurture (and assortative mating) made little contribution to the SNP-heritability of *avMSE*.

## Discussion

Time spent outdoors, reading or playing video games is associated with the risk of myopia and is also correlated within siblings, suggesting a familial element to these behaviours [42]. Furthermore, a child’s refractive error PGS and the number of parents with myopia were reported to be independent predictors of myopia in children, indicating that parents may pass on myopia-predisposing environments as well as myopia-predisposing alleles to their children [43, 44]. Gene-environment correlation (expected in genetic nurture) has also been shown in the development of myopia [44]. A history of smoking by paternal grandmothers has been reported to be associated with early-onset myopia [45]. Epidemiological studies have consistently demonstrated a strong association between educational attainment and refractive error [46]. Indeed, Mendelian randomization and regression discontinuity analyses have suggested that this relationship may be causal [11, 12, 34]. These prior links between family environment, education and myopia led us to hypothesize that genetic nurture would play an important role in refractive error development, and that, if so, genetic nurture may upwardly bias estimates of the SNP-heritability for refractive error [47]. The key finding in this study was a lack of support for a contribution from genetic nurture to the genetics of refractive error. The lower bound of the 95% confidence interval for the within-family genetic contribution $\alpha _1^{wf}$ was 0.152, which, in comparison to the point estimate of 0.250, suggests genetic nurture contributed at most 40% (0.250–0.152/0.250) of the SNP-heritability of refractive error in the UK Biobank sample. Analyses in larger samples will be needed to gain more precise estimates.

The above-chance correlation of polygenic scores within pairs of siblings suggested that assortative mating may be present for both educational attainment and refractive error, which supports past findings [41, 48, 49]. Our estimate of the SNP-heritability for educational attainment ($h_{{{{{\rm{SNP}}}}}}^2$ ≈ 0.17) is in line with previous estimates [16, 50]. Our SNP-heritability estimate for refractive error ($h_{{{{{\rm{SNP}}}}}}^2$ ≈ 0.22) is lower than previous estimates [7, 47], but the precision of the current assessment was limited by the small sample size. It was notable, in this study, that only about 25% of the SNP-heritability of educational attainment was attributed to alleles transmitted from parents to children ($\alpha _1^{wf} = 0.04$ vs. $\alpha _1^{nwf} = 0.17$), with the rest being attributed to genetic nurture or assortative mating. Previous studies have suggested the contribution from transmitted alleles to educational attainment to be approximately 50% rather than 25% [16, 50]. One potential reason for this is that previous studies may not have fully accounted for attenuation bias resulting when performing a PGS-based mediation analysis [31], whereas we used an instrumented PGS to minimize this bias. Additional factors, such as differences between study populations and phenotype definitions, may also be relevant.

Studies of siblings offer distinct benefits compared to studying genetic risk in samples of unrelated individuals. Case-control association studies in unrelated samples rely on the principle that disease risk-increasing alleles will be over-represented in cases compared to controls. However, three other phenomena can also cause a significant over-representation of alleles in cases: population stratification, assortative mating and genetic nurture [40, 48]. Analyses of siblings, on the other hand, can be designed to be free from these biases [41]. In the current study, our finding that $\alpha _1^{nwf}$ ≈ $\alpha _1^{wf}$ for refractive error suggested that population stratification, assortative mating and genetic nurture made little contribution to the SNP-heritability of refractive error.

Strengths of the current work were the highly standardized method for measuring refractive error, use of powerful PGS constructs implemented in an instrumental variable framework [31, 33]. The main weakness of the current work was the limited sample available for the analysis of refractive error (*n* = 1944 pairs of siblings), leading to wide confidence intervals for the key parameters of $\alpha _1^{wf}$and $\alpha _1^{nwf}$ and to insufficient power to examine genetic nurture effects for specific SNPs strongly associated with refractive error–as previously applied to investigate specific variants associated with birth weight [30]. Although autorefraction is considered the gold standard method for measuring refractive error in research studies [51], differences between autorefraction readings and manifest refraction would have contributed to the imprecision of the polygenic scores for refractive error.

When considering these new results, it is important to bear in mind that our analyses were conducted in a group of participants who grew up in the United Kingdom during the 1940s to 1980s. As such, our findings do not rule out a greater role for genetic nurture in more recent birth cohorts, or in communities with differing lifestyle-related risk factors, such as urban regions in East and South-East Asia where the prevalence of myopia is much higher than the United Kingdom [1]. Given these caveats, the current observation that genetic nurture makes a limited contribution to refractive error has several implications. First, it suggests that genetic variants identified in GWASs for refractive error are likely to exert their effects in probands rather in their parent, highlighting the genes that are potential therapeutic targets in children; this point has previously been assumed, but without any supporting evidence [5, 7]. Second, the evidence that genetic variants act directly in probands suggests that polygenic scores for predicting children at high risk of myopia may have greater clinical utility than they would otherwise. For instance, selecting a child’s treatment based on his/her PGS may be both more effective and more ethically justified, if genetic risk for myopia is intrinsic rather than a function of the environment. More widely, it will be of interest to investigate if genetic nurture contributes to other heritable ocular traits such as astigmatism and glaucoma [52, 53].

In summary, in this study, we obtained minimally-biased estimates of the genetic contribution to refractive error and educational attainment; specifically, free from bias due to genetic nurture and assortative mating. Building on prior work, we estimated that ~25% of the SNP-heritability of educational attainment was attributed to alleles transmitted from parents to children and that the remaining ~75% occurred as a result of genetic nurture and assortative mating. In stark contrast, our results suggested little contribution from genetic nurture to the SNP-heritability of refractive error. While we did find evidence of assortative mating for refractive error, there was no evidence that this appreciably inflated its SNP-heritability estimate. We conclude that, whereas the genetic contribution to educational attainment is in large part inherited through the environment, the genetic contribution to refractive error occurs mainly through direct parent-to-child transmission of alleles. Our findings validate the assumption that genetic variants associated with refractive error highlight potential therapeutic targets.

## Supplementary information

Supplementary Material

## Data Availability

Individual-level data from UK Biobank can be accessed by applying to the UK Biobank (https://www.ukbiobank.ac.uk/enable-your-research/apply-for-access).

## Code availability

R code for implementing the statistical analysis is provided in Supplementary Methods.
