# Supplementary material for: Assessing the contribution of genetic nurture to refractive error
Source: Eur J Hum Genet. 2022 May 27;30(11):1226–32. doi: 10.1038/s41431-022-01126-6 (PMC9626539; doi:10.1038/s41431-022-01126-6)
Supplement: Supplementary file 1 — Supplementary Material [file 41431_2022_1126_MOESM1_ESM.docx]

**Supporting Information**

**Assessing the contribution of genetic nurture to refractive error**

Membership of the UK Biobank Eye and Vision Consortium

Supplementary Table S1. Parameter estimates for all variables included in EduYears analysis.

Supplementary Table S2. Parameter estimates for all variables included in avMSE analysis.

Supplementary Figure S1. Selection of siblings.

Supplementary Methods. R code to replicate the analysis.

**Membership of the UK Biobank Eye and Vision Consortium**

Naomi Allen^2^, Tariq Aslam^3^, Denize Atan^5^, Sarah A. Barman^4^, Jenny H. Barrett^6^, Paul Bishop^3^ , Graeme Black^3^, Tasanee Braithwaite^7^, Roxana Carare^8^, Usha Chakravarthy^9^, Michelle Chan^10^, Sharon Chua^11^, Alexander Day^10^, Parul Desai^10^, Bal Dhillon^12^, Andrew Dick^5^, Alexander Doney^13^, Cathy Egan^10^, Sarah Ennis^8^, Paul Foster^11^, Marcus Fruttiger^11^, John Gallacher^14^, David Garway-Heath^11^, Jane Gibson^8^, Jeremy Guggenheim^1^, Chris Hammond^7^, Alison Hardcastle^11^, Simon Harding^15^, Ruth Hogg^9^, Pirro Hysi^7^, Pearse Keane^11^, Sir Peng Tee Khaw^11^, Anthony Khawaja^10^, Gerassimos Lascaratos^10^, Thomas Littlejohns^2^, Andrew Lotery^8^, Phil Luthert^11^, Tom Macgillivray^12^, Sarah Mackie - University of Leeds, Bernadette Mcguinness^9^, Gareth Mckay^9^, Martin Mckibbin^6^, Tony Moore^11^, James Morgan^1^, Eoin O'sullivan^7^, Richard Oram^16^, Chris Owen^17^, Praveen Patel^10^, Euan Paterson^9^, Tunde Peto^9^, Axel Petzold^18^, Nikolas Pontikos^11^, Jugnoo Rahi^19^, Alicja Rudnicka^17^, Naveed Sattar^20^, Jay Self^8^, Panagiotis Sergouniotis^3^, Sobha Sivaprasad^10^, David Steel^21^, Irene Stratton^22^, Nicholas Strouthidis^10^, Cathie Sudlow^23^, Zihan Sun^11^, Robyn Tapp^24^, Dhanes Thomas^10^, Emanuele Trucco^25^, Adnan Tufail^10^, Ananth Viswanathan^10^, Veronique Vitart^26^, Mike Weedon^16^, Katie Williams^7^, Cathy Williams^5^, Jayne Woodside^27^, Max Yates^28^, Jennifer Yip^29^, Yalin Zheng^15^.

Affiliations: 2. Nuffield Department of Population Health, University of Oxford., Oxford, UK. 3. Faculty of Biology, Medicine and Health, University of Manchester, Manchester, UK. 4. Faculty of Science, Engineering and Computing, Kingston University, London, UK. 6. School of Medicine, University of Leeds, Leeds, UK. 7. King's College London, London, UK. 8. School of Medicine, University of Southampton, Southampton, UK. 9. Centre for Public Health, Queen's University Belfast, Belfast, UK. 10. Moorfields Eye Hospital NHS Foundation Trust, London, UK. 11. UCL Institute of Ophthalmology, London, UK. 12. Centre for Clinical Brain Sciences, Division of Health Sciences, University of Edinburgh, Edinburgh, UK. 13. School of Medicine, University of Dundee, Dundee, UK. 14. Department of Psychiatry, University of Oxford., Oxford, UK. 15. Institute of Life Course and Medical Sciences, University of Liverpool, Liverpool, UK. 16. Exeter Medical School, University of Exeter, Exter, UK. 17. Population Health Research Institute, St George's University of London, London, UK. 18. UCL Institute of Neurology, London, UK. 19. UCL Institute of Child Health, London, UK. 20. Institute of Cardiovascular & Medical Sciences, University of Glasgow, Glasgow, UK. 21. Institute of Genetic Medicine, Newcastle University, Newcastle, UK. 22. Gloucestershire Hospitals NHS Foundation Trust, Cheltenham, UK 23. The Usher Institute, Centre for Clinical Brain Sciences, and Centre for Genomic and Experimental Medicine, University of Edinburgh, Edinburgh, UK. 24. Research Centre for Intelligent Healthcare, University of Coventry, Coventry, UK. 25. School of Science and Engineering, University of Dundee, Dundee, UK. 26. MRC Human Genetics Unit, University of Edinburgh, Edinburgh, UK. 27. School of Medicine, Dentistry and Biomedical Sciences, Queen's University Belfast, Belfast, UK. 28. Norwich Medical School, University of East Anglia, Norwich, UK. 29. International Centre for Evidence on Disability, London, UK.

**Supplementary Table S1. Parameter estimates for all variables included in *EduYears* analysis.**

| **Outcome: *EduYears*** | **Non-within-family model** | | |  | **Within-family model:** | | |
| --- | --- | --- | --- | --- | --- | --- | --- |
|  | **Estimate** | **SE** | ***P*-value** |  | **Estimate** | **SE** | ***P*-value** |
| Intercept | 0.045 | 0.032 | 1.62E-01 |  | - | - | - |
| Proband PGS^a^ | 0.585 | 0.014 | <1.00E-100 |  | 0.279 | 0.019 | 1.15E-47 |
| Sex | -0.091 | 0.015 | 9.32E-10 |  | -0.100 | 0.012 | 1.60E-17 |
| Age (years)^b^ | -0.200 | 0.008 | <1.00E-100 |  | -0.119 | 0.011 | 1.37E-27 |
| Age^2 (years) ^b^ | -0.047 | 0.009 | 2.53E-08 |  | -0.028 | 0.008 | 2.47E-04 |
| Genotyping array | 0.091 | 0.024 | 1.32E-04 |  | 0.065 | 0.018 | 2.37E-04 |
| PC1^b^ | -0.013 | 0.009 | 1.43E-01 |  | 0.004 | 0.010 | 6.82E-01 |
| PC2^b^ | -0.009 | 0.008 | 2.61E-01 |  | 0.010 | 0.009 | 2.62E-01 |
| PC3^b^ | 0.005 | 0.009 | 5.75E-01 |  | 0.006 | 0.009 | 5.23E-01 |
| PC4^b^ | -0.038 | 0.012 | 1.03E-03 |  | 0.003 | 0.017 | 8.53E-01 |
| PC5^b^ | 0.017 | 0.010 | 7.49E-02 |  | 0.019 | 0.031 | 5.37E-01 |
| PC6^b^ | 0.001 | 0.008 | 9.14E-01 |  | 0.002 | 0.009 | 8.03E-01 |
| PC7^b^ | 0.004 | 0.008 | 6.02E-01 |  | 0.004 | 0.011 | 7.44E-01 |
| PC8^b^ | 0.002 | 0.009 | 8.30E-01 |  | 0.019 | 0.012 | 1.02E-01 |
| PC9^b^ | -0.014 | 0.008 | 7.42E-02 |  | -0.027 | 0.020 | 1.67E-01 |
| PC10^b^ | 0.002 | 0.008 | 8.28E-01 |  | 0.002 | 0.011 | 8.62E-01 |
| ^a^ Genetic contribution on heritability scale = (PGS beta coefficient^2)*0.509, where 0.509 = cor(${PGS}_{\left( m=1 \right)},{PGS}_{\left( m=2 \right)}$) for *EduYears*.  ^b^ All continuous predictor variables were standardized to have a mean of zero and a standard deviation of one. | | | | | | | |

**Supplementary Table S2. Parameter estimates for all variables included in *avMSE* analysis.**

| **Outcome: *avMSE*** | **Non-within-family model** | | |  | **Within-family model:** | | |
| --- | --- | --- | --- | --- | --- | --- | --- |
|  | **Estimate** | **SE** | ***P*-value** |  | **Estimate** | **SE** | ***P*-value** |
| Intercept | 0.032 | 0.098 | 7.46E-01 |  | - | - | - |
| Proband PGS^a^ | 0.821 | 0.086 | 6.36E-21 |  | 0.889 | 0.100 | 1.32E-18 |
| Sex | 0.037 | 0.049 | 4.52E-01 |  | 0.024 | 0.042 | 5.65E-01 |
| Age (years)^b^ | 0.249 | 0.026 | 9.88E-21 |  | 0.178 | 0.042 | 2.01E-05 |
| Age^2 (years) ^b^ | 0.074 | 0.028 | 8.37E-03 |  | 0.063 | 0.029 | 2.69E-02 |
| Genotyping array | -0.128 | 0.070 | 6.70E-02 |  | -0.113 | 0.067 | 9.28E-02 |
| PC1^b^ | 0.001 | 0.030 | 9.69E-01 |  | 0.055 | 0.040 | 1.71E-01 |
| PC2^b^ | 0.030 | 0.024 | 2.23E-01 |  | 0.047 | 0.031 | 1.27E-01 |
| PC3^b^ | 0.018 | 0.029 | 5.37E-01 |  | 0.050 | 0.035 | 1.48E-01 |
| PC4^b^ | -0.010 | 0.039 | 8.06E-01 |  | 0.032 | 0.065 | 6.25E-01 |
| PC5^b^ | 0.006 | 0.032 | 8.56E-01 |  | 0.076 | 0.115 | 5.08E-01 |
| PC6^b^ | -0.002 | 0.027 | 9.44E-01 |  | -0.009 | 0.035 | 7.85E-01 |
| PC7^b^ | 0.012 | 0.027 | 6.60E-01 |  | -0.032 | 0.039 | 4.22E-01 |
| PC8^b^ | 0.001 | 0.029 | 9.78E-01 |  | 0.014 | 0.043 | 7.38E-01 |
| PC9^b^ | -0.008 | 0.029 | 7.94E-01 |  | -0.059 | 0.069 | 3.90E-01 |
| PC10^b^ | 0.024 | 0.028 | 4.03E-01 |  | 0.039 | 0.039 | 3.22E-01 |
| ^a^ Genetic contribution on heritability scale = (PGS beta coefficient^2)*0.316 where 0.316 = cor(${PGS}_{\left( m=1 \right)},{PGS}_{\left( m=2 \right)}$) for *avMSE*.  ^b^ All continuous predictor variables were standardized to have a mean of zero and a standard deviation of one. | | | | | | | |

**Supplementary Figure S1. Selection of siblings.** Siblings (red data points) were identified as individuals with a kinship coefficient between 0.177–0.354 and a proportion of markers for which no alleles were shared identical-by-state (IBS0) between 0.0015–0.012. Two outlier pairs whose kinship vs. IBS0 pattern did not cluster with the other sibling pairs were excluded (blue data points).


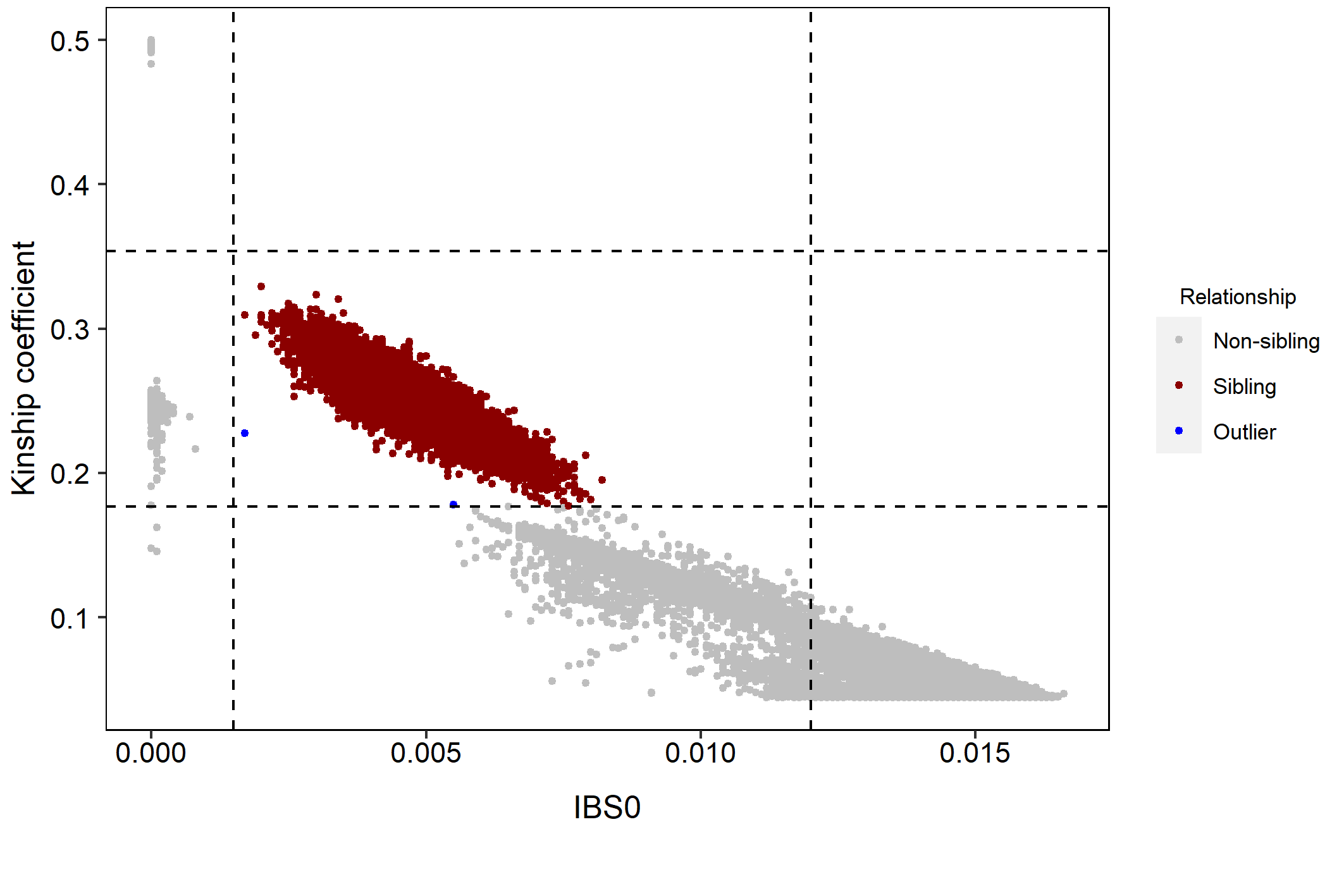


**Supplementary Methods:**

R code to replicate the analyses.

library(data.table)

library(plm)

library(ivpack)

rm(list=ls())

get_table <- function(mydata,myrow,mycor){

mydata <- as.data.frame(mydata)

myrow <- as.integer(myrow)

mycor <- as.numeric(mycor)

a <- mydata[myrow,1] # estimate

b <- mydata[myrow,2] # se

c <- mydata[myrow,4] # p

d <- a - (1.96*b)

e <- a + (1.96*b)

R0 <- row.names(mydata)[myrow]

R1 <- round((a^2)*mycor,3)

R2 <- paste("(", sign(d)*round((d^2)*mycor,3), " to ", sign(e)*round((e^2)*mycor,3), ")", sep="")

R3 <- c

R <- c(R0,R1,R2,R3)

return(R)

}

# Data files

# ----------

mydir=[path to local directory]

file_avMSE_siblings <- paste(mydir,"genetic_nuture/results/outfile_avMSE_siblings.txt",sep="")

file_EduYears_siblings <- paste(mydir,"genetic_nuture/results/outfile_EduYears_siblings.txt",sep="")

file_all_scores <- paste(mydir,"genetic_nuture/results/scores_all_PRS.txt",sep="")

# file_avMSE_siblings: data frame with data for 3888 individuals from 1944 sibpairs.

# ----------------------------------------------------------------------------------

# IID : Family ID code

# FID : Individual ID code

# Sex_matched : Sex (1=M, 2=F)

# Geno_array : Genotyping array (1= UK BiLEVE Axiom, 2=UK Biobank Axiom)

# NewAge : Age (standardised)

# NewAge2 : Age squared (standardised)

# NewPC1 : PC1 (standardised)

# NewPC2-NewPC10 : PC2 - PC10 (all standardised)

# avMSE : Refractive error averaged between right and left eyes (D)

# sibpairID : Sibpair ID number

# file_EduYears_siblings: data frame with data for 36292 individuals from 18146 sibpairs.

# ----------------------------------------------------------------------------------

# IID : Family ID code

# FID : Individual ID code

# Sex_matched : Sex (1=M, 2=F)

# Geno_array : Genotyping array (1= UK BiLEVE Axiom, 2=UK Biobank Axiom)

# NewAge : Age (standardised)

# NewAge2 : Age squared (standardised)

# NewPC1 : PC1 (standardised)

# NewPC2-NewPC10 : PC2 - PC10 (all standardised)

# EduYears : Years spent in full-time education

# sibpairID : Sibpair ID number

# file_EduYears_siblings: data frame with data for 442000 individuals of EUR ancestry.

# ------------------------------------------------------------------------------------

# IID : Family ID code

# FID : Individual ID code

# avMSE_PRS1 : First polygenic risk score for avMSE

# avMSE_PRS2 : Second polygenic risk score for avMSE

# EduYears_PRS1 : First polygenic risk score for EduYears

# EduYears_PRS2 : second polygenic risk score for EduYears

# Merge data files

# ----------------

data1 <- as.data.frame(fread(file=file_avMSE_siblings, header=TRUE))

data2 <- as.data.frame(fread(file=file_EduYears_siblings, header=TRUE))

data3 <- as.data.frame(fread(file=file_all_scores, header=TRUE))

data3$avMSE_sPRS1 <- scale(data3$avMSE_PRS1)

data3$avMSE_sPRS2 <- scale(data3$avMSE_PRS2)

data3$EduYears_sPRS1 <- scale(data3$EduYears_PRS1)

data3$EduYears_sPRS2 <- scale(data3$EduYears_PRS2)

data1$savMSE <- scale(data1$avMSE)

data2$sEduYears <- scale(data2$EduYears)

data4 <- merge(data1,data3[,c("IID","avMSE_sPRS1","avMSE_sPRS2")],by="IID")

data5 <- merge(data2,data3[,c("IID","EduYears_sPRS1","EduYears_sPRS2")],by="IID")

# ==================

# EDUCATION ANALYSIS

# ==================

EduYears_sample_size <- dim(data5)[1]

data5$myrand <- runif(EduYears_sample_size, min=0, max=1)

dataSIBEDU <- data5[order(data5$sibpairID,data5$myrand),] # Randomly select one sibling as Sib A

dataSIBEDU$AB <- rep(c(1,2),(EduYears_sample_size/2))

dataSIBEDU$sibsEduYears <- NA

dataSIBEDU[which(dataSIBEDU$AB==1),]$sibsEduYears <- dataSIBEDU[which(dataSIBEDU$AB==2),]$sEduYears

dataSIBEDU[which(dataSIBEDU$AB==2),]$sibsEduYears <- dataSIBEDU[which(dataSIBEDU$AB==1),]$sEduYears

dataSIBEDU$sibEduYears_sPRS1 <- NA

dataSIBEDU[which(dataSIBEDU$AB==1),]$sibEduYears_sPRS1 <- dataSIBEDU[which(dataSIBEDU$AB==2),]$EduYears_sPRS1

dataSIBEDU[which(dataSIBEDU$AB==2),]$sibEduYears_sPRS1 <- dataSIBEDU[which(dataSIBEDU$AB==1),]$EduYears_sPRS1

dataSIBEDU$sibEduYears_sPRS2 <- NA

dataSIBEDU[which(dataSIBEDU$AB==1),]$sibEduYears_sPRS2 <- dataSIBEDU[which(dataSIBEDU$AB==2),]$EduYears_sPRS2

dataSIBEDU[which(dataSIBEDU$AB==2),]$sibEduYears_sPRS2 <- dataSIBEDU[which(dataSIBEDU$AB==1),]$EduYears_sPRS2

EduRes2 <- as.data.frame(matrix(nrow=2,ncol=4))

names(EduRes2) <- c("Parameter","Estimate","95CI","P")

dataTEST <- dataSIBEDU[which(dataSIBEDU$AB==2),]

mycor <- as.numeric(cor(dataTEST$EduYears_sPRS1,dataTEST$EduYears_sPRS2))

estimates_NWF <- robust.se(ivreg(sEduYears ~ EduYears_sPRS1 + Sex_matched + NewAge + NewAge2 + Geno_array + NewPC1 + NewPC2 + NewPC3 +

NewPC4 + NewPC5 + NewPC6 + NewPC7 + NewPC8 + NewPC9 + NewPC10 |

EduYears_sPRS2 + Sex_matched + NewAge + NewAge2 + NewPC1 + Geno_array + NewPC2 + NewPC3 +

NewPC4 + NewPC5 + NewPC6 + NewPC7 + NewPC8 + NewPC9 + NewPC10,

x = TRUE, data = dataSIBEDU[which(dataSIBEDU$AB==2),]))

modWF <- plm(sEduYears ~ EduYears_sPRS1 + Sex_matched + NewAge + NewAge2 + Geno_array + NewPC1 + NewPC2 + NewPC3 +

NewPC4 + NewPC5 + NewPC6 + NewPC7 + NewPC8 + NewPC9 + NewPC10 |

EduYears_sPRS2 + Sex_matched + NewAge + NewAge2 + Geno_array + NewPC1 + NewPC2 + NewPC3 +

NewPC4 + NewPC5 + NewPC6 + NewPC7 + NewPC8 + NewPC9 + NewPC10,

data = dataSIBEDU, index = "sibpairID", model = "within", inst.method = "bvk")

estimates_WF <- coeftest(modWF, vcov=vcovHC(modWF,type="HC0", cluster="group"))

EduRes2[1,] <- get_table(estimates_NWF[1:2,1:4], 2, mycor)

EduRes2[1,1] <- "Non-within-family"

EduRes2[2,] <- get_table(estimates_WF[1:2,1:4], 1, mycor)

EduRes2[2,1] <- "Within-family"

EduRes2

# Parameter Estimate 95CI P

#1 Non-within-family 0.174 (0.158 to 0.191) 0

#2 Within-family 0.04 (0.03 to 0.051) 1.14722461978666e-47

# =========================

# REFRACTIVE ERROR ANALYSIS

# =========================

set.seed(9876)

avMSE_sample_size <- dim(data4)[1]

data4$myrand <- runif(avMSE_sample_size, min=0, max=1)

dataSIBMSE <- data4[order(data4$sibpairID,data4$myrand),] # Randomly select one sibling as Sib A

dataSIBMSE$AB <- rep(c(1,2),(avMSE_sample_size/2))

dataSIBMSE$sibsavMSE <- NA

dataSIBMSE[which(dataSIBMSE$AB==1),]$sibsavMSE <- dataSIBMSE[which(dataSIBMSE$AB==2),]$savMSE

dataSIBMSE[which(dataSIBMSE$AB==2),]$sibsavMSE <- dataSIBMSE[which(dataSIBMSE$AB==1),]$savMSE

dataSIBMSE$sibavMSE_sPRS1 <- NA

dataSIBMSE[which(dataSIBMSE$AB==1),]$sibavMSE_sPRS1 <- dataSIBMSE[which(dataSIBMSE$AB==2),]$avMSE_sPRS1

dataSIBMSE[which(dataSIBMSE$AB==2),]$sibavMSE_sPRS1 <- dataSIBMSE[which(dataSIBMSE$AB==1),]$avMSE_sPRS1

dataSIBMSE$sibavMSE_sPRS2 <- NA

dataSIBMSE[which(dataSIBMSE$AB==1),]$sibavMSE_sPRS2 <- dataSIBMSE[which(dataSIBMSE$AB==2),]$avMSE_sPRS2

dataSIBMSE[which(dataSIBMSE$AB==2),]$sibavMSE_sPRS2 <- dataSIBMSE[which(dataSIBMSE$AB==1),]$avMSE_sPRS2

dataTEST <- dataSIBMSE[which(dataSIBMSE$AB==2),]

MSERes2 <- as.data.frame(matrix(nrow=2,ncol=4))

names(MSERes2) <- c("Parameter","Estimate","95CI","P")

mycor <- as.numeric(cor(dataTEST$avMSE_sPRS1,dataTEST$avMSE_sPRS2))

estimates_NWF <- robust.se(ivreg(savMSE ~ avMSE_sPRS1 + Sex_matched + NewAge + NewAge2 + Geno_array + NewPC1 + NewPC2 + NewPC3 +

NewPC4 + NewPC5 + NewPC6 + NewPC7 + NewPC8 + NewPC9 + NewPC10 |

avMSE_sPRS2 + Sex_matched + NewAge + NewAge2 + NewPC1 + Geno_array + NewPC2 + NewPC3 +

NewPC4 + NewPC5 + NewPC6 + NewPC7 + NewPC8 + NewPC9 + NewPC10,

x = TRUE, data = dataSIBMSE[which(dataSIBMSE$AB==2),] ))

modWF <- plm(savMSE ~ avMSE_sPRS1 + Sex_matched + NewAge + NewAge2 + Geno_array + NewPC1 + NewPC2 + NewPC3 +

NewPC4 + NewPC5 + NewPC6 + NewPC7 + NewPC8 + NewPC9 + NewPC10 |

avMSE_sPRS2 + Sex_matched + NewAge + NewAge2 + Geno_array + NewPC1 + NewPC2 + NewPC3 +

NewPC4 + NewPC5 + NewPC6 + NewPC7 + NewPC8 + NewPC9 + NewPC10,

data = dataSIBMSE, index = "sibpairID", model = "within", inst.method = "bvk")

estimates_WF <- coeftest(modWF, vcov=vcovHC(modWF,type="HC0", cluster="group"))

MSERes2[1,] <- get_table(estimates_NWF[1:2,1:4], 2, mycor)

MSERes2[1,1] <- "Non-within-family"

MSERes2[2,] <- get_table(estimates_WF[1:2,1:4], 1, mycor)

MSERes2[2,1] <- "Within-family"

MSERes2

# Parameter Estimate 95CI P

#1 Non-within-family 0.213 (0.134 to 0.31) 6.36057797594402e-21

#2 Within-family 0.25 (0.152 to 0.372) 1.32189423423581e-18
